# Polysaccharides from Garlic Protect against Liver Injury in DSS-Induced Inflammatory Bowel Disease of Mice via Suppressing Pyroptosis and Oxidative Damage

**DOI:** 10.1155/2022/2042163

**Published:** 2022-08-16

**Authors:** Xinyi Zhan, Weijie Peng, Zhuqiang Wang, Xin Liu, Weibo Dai, Quanxi Mei, Xianjing Hu

**Affiliations:** ^1^Pharmacology Laboratory, Zhongshan Hospital, Guangzhou University of Chinese Medicine, Zhongshan 528401, China; ^2^Shenzhen Baoan Authentic TCM Therapy Hospital, Shenzhen 518101, China; ^3^Guangdong Provincial Key Laboratory of Research and Development of Natural Drugs, And School of Pharmacy, Guangdong Medical University, Dongguan 523808, China

## Abstract

Inflammatory bowel disease (IBD), a widespread intestinal disease threatening human health, is commonly accompanied by secondary liver injury (SLI). Pyroptosis and oxidative stress act as an important role underlying the pathophysiology of SLI, during which a large number of proinflammatory cytokines and oxidative intermediates can be produced, thereby causing the liver severely damaged. Suppression of pyroptosis and oxidative damage can be considered one of the critical strategies for SLI therapy. Garlic, a natural food with eatable and medicinal functions, is widely used in people's daily life. There is no study about the alleviation of garlic against IBD accompanied with SLI. This study is aimed at investigating the efficacy of the polysaccharides from garlic (PSG) in treating IBD and SLI, as well as its pharmacological mechanism. The results showed that PSG significantly alleviated dextran sulfate sodium-induced IBD determined by evaluating the bodyweight loss, disease activity index, colon length, and colonic pathological examination of mice. PSG significantly reduced the colonic inflammation by reversing the levels of myeloperoxidase, diamine oxidase activity, iNOS, and COX2 and strengthened the intestinal barrier by increasing the expressions of ZO1, occludin, and MUC2 of IBD mice. Furthermore, PSG strongly alleviated SLI determined by assessing the liver morphological change, liver index, levels of ALT and AST, and liver pathological change of mice. Mechanically, PSG reduced the high levels of LPS, IL-1*β*, IL18, NLRP3, gasdermin D, caspase 1, ASC, TLR4, MyD88, NF-*κ*B, phospho-NF-*κ*B, while it increased IL-10 in the livers of mice, indicating that PSG alleviated SLI by suppressing inflammation and pyroptosis. Additionally, PSG significantly inhibited the oxidative damage in the liver tissues of SLI mice by reducing the levels of ROS, MDA, Keap-1, 8-OHDG, and phospho-H2AX and increasing the levels of GPX4, SOD2, HO1, NQO1, and Nrf2. These findings suggested that the garlic polysaccharides could be used to treat IBD accompanied with SLI in humans.

## 1. Introduction

Inflammatory bowel disease (IBD), a widely spreading disease, is characterized by body weight loss, severe diarrhea, abdominal cramps, and even rectal bleeding [1]. According to the epidemiological survey, more than 3.6 million people are suffering from IBD around the world and the number is still rising rapidly [2]. The Center for Disease Control and Prevention of China reported that ~350,000 IBD cases in China occurred in the past ten years and the number was predicted to be 1.5 million by 2025 [3]. Presently, several immunosuppressive agents and anti-inflammatory drugs, such as corticosteroid, 5-aminosalicylic acid (5-ASA), and salicylazosulfapyridine (SASP), are the main drugs used in the clinic to control the symptoms of IBD [4]. However, a long-time use of these drugs would induce severe side effects, greatly limiting their clinical application [5]. Monoclonal antibodies targeting proinflammatory cytokines, such as adalimumab (a kind of TNF-*α* antibody), are also commonly used in the clinic for the advanced stage of IBD [6], while the efficacies of these antibody drugs are not so satisfactory because multiple inflammatory cytokines and signaling pathways are involved during IBD development [7], and just targeting only one cytokine is not sufficient for treatment. Therefore, it is still urgently demanded to develop new agents with high efficacy, low side effects, and multitargets to treat IBD.

Continuous inflammation response is involved in the pathogenesis of IBD [8], and a series of proinflammatory cytokines are excessively produced, such as IL-6, TNF-*α*, and IFN-*γ* [9]. In the clinic, various complicating diseases have been reported to be associated with IBD [10, 11], of which the secondary liver injury (SLI) is one of the most common that would be probably aggravated and lead to chronic hepatopathy [12]. In the pathological status of IBD, as the intestinal mucosa is damaged and intestinal permeability is increased, the intestinal bacteria along with their harmful products, such as lipopolysaccharides (LPS), TNF-*α*, and IL-6, will enter the liver through the portal vein, which would cause a liver inflammatory reaction and aggravate liver injury [13]. IBD treatment is considered to be a good strategy for protecting from liver injury mediated by regulating the hepatoenteric circulation.

It is reported that pyroptosis acts as an important mechanism in the evolution of IBD and SLI, during which the cytokines of IL-1*β* and IL-18 can be cleaved and activated into mature IL-1*β* and IL-18, thereby aggravating the inflammatory injury of tissues [14]. Oxidative stress is also regarded as an important mechanism underlying the pathophysiology of IBD and SLI, during which excessive reactive oxygen and nitrogen species would be produced and then promote chronic inflammation development via strengthening the TLR4/NF-*κ*B pathway [15]. Moreover, oxygen-free radicals can cause damage to DNA, protein, and other biological macromolecules, resulting in tissues damaged [16]. The study also reported that oxidative stress would increase the inflammatory cytokines and enhance the infiltration of inflammatory cells [17]. Therefore, the levels of antioxidant or oxidative injury biomarkers are usually recognized to reflect the disease severity and the extent of intestinal inflammation of IBD and SLI, and agents with good efficacy in suppressing oxidative injury can be regarded as promising remedies for treating IBD accompanied with SLI.

Polysaccharide is a class of high-molecular carbohydrates formed by dehydration condensation of multiple monosaccharide molecules, widely existing in nature, and has the characteristics of multiple targets and little toxicity [18]. Presently, more than 400 polysaccharides have been found to possess the bioactivities of anti-inflammation, antioxidation, antitumor, maintenance of intestinal homeostasis, and so on, such as *Chrysanthemum morifolium* Ramat. [19], *Camellia sinensis* L. [20], and *Dendrobium officinale* Kimura et Migo [21]. Garlic (*Allium sativum* L.) is a kind of natural food with both eatable and medicinal functions, which has been served as a flavoring agent used in people's daily life and also as a traditional Chinese medicine for thousands of years to treat a series of diseases, such as coughs, colds, diabetes, obesity, tuberculosis, minor vascular disorders, hyperpiesia, and kidney and liver injury [22]. However, little information is known about the therapeutic function of its polysaccharides (PSG) against IBD-accompanied SLI and its underlying mechanism. This study is designed to investigate the alleviation of DSS-induced IBD and SLI in mice, as well as uncover the pharmacological mechanism.

## 2. Materials and Methods

### 2.1. Materials and Reagents

Dextran sulfate sodium (DSS, MW = 36 − 50 kDa, S5036, 9011-18-1) was obtained from MP Biomedicals Inc. (Irvine, CA, USA). 5-Aminosalicylic acid (5-ASA) (98% of purity, CAS. 89-57-6) was purchased from Meilun Biotechnology Co., Ltd. (Dalian, China). Alanine transaminase (ALT, 20210428) and aspartate aminotransferase (AST, 20210427) assay kits were purchased from Nanjing Jiancheng Bioengineering Institute (Nanjing, China). MPO (#m1002070-2) and DAO (#m1002199-2) assay kits were purchased from Shanghai MLBIO Biotechnology Co., Ltd. (Shanghai, China). ELISA kits, including LPS (261210519), IL-1*β* (370210519), IL-10 (371210513), IL-18 (375210406), MDA (417210607), and 8-OHDG (108220412), were purchased from Tianjin Anoric Biotechnology Co., Ltd. (Tianjin, China). ROS (MM-43700M1) assay kit was obtained from Meimian Biology (Jiangsu, China). Primary antibodies against ZO1 (AF5145), occludin (DF7504), MyD88 (AF5195), HO1 (AF5393), NQO1 (DF6437), Keap1 (AF5266), gasdermin D (AF4012), caspase 1 (AF5418), SOD2 (AF5144), GPX4 (DF6701), and *β*-actin (T0022) were obtained from Affinity Biosciences Ltd. (OH, USA). Primary antibodies against iNOS (13120), COX2 (12282), NF-*κ*B (8242s), and phospho-NF-*κ*B (3033t) were obtained from Cell Signal Technology Inc. (Boston, USA). Primary antibodies against Nrf2 (A0674), NLRP3 (A5652), ASC (A1170), and phospho-histone H2AX-S139 (AP0099) were obtained from ABclonal Biotechnology Co., Ltd. (Wuhan, China), and TLR4 (293072) was from Santa Cruz Biotechnology Inc. (California, USA), respectively. The material of garlic was “Jinxiang garlic,” obtained from Jinxiang County, Jining, China.

### 2.2. Preparation and Characterization Analysis of PSG

**Figure 1:**
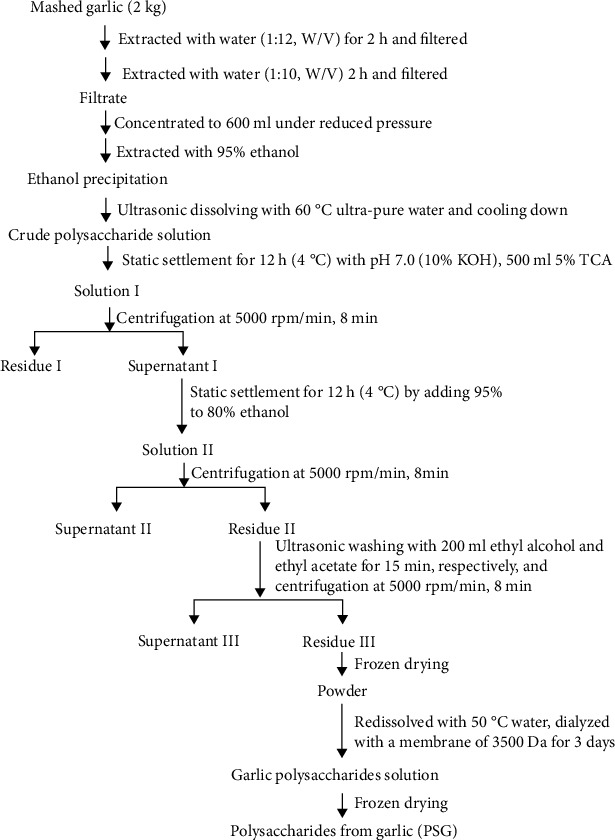
Procedure for the extraction of polysaccharides from garlic (PSG).

The preparation of PSG was performed according to the procedure shown in Figure 1. The total sugar content of PSG was determined by the phenol-sulfuric acid method, and protein content was determined by a protein assay kit. The average molecular weight of PSG was determined by gel permeation chromatography (GPC). The compositions of monosaccharides and uronic acid in PSG were analyzed by the trifluoroacetic acid hydrolysis and the high-performance liquid chromatography (HPLC) method. The HPLC chromatographic conditions were as follows: Agilent 1100 C18 column (4.6 mm × 250 mm, 5 *μ*m); 90 mmol/L sodium phosphate buffer was used as mobile phase A, and 100% acetonitrile was used as mobile phase B; gradient: 13% B→16% B (0–9 min), 16% B→19% B (9–20 min), 19% B→40% B (20–23 min), 40% B (23–25 min), 40% B→13% B (25–25.5 min), and 13% B (25.5–30 min); flow rate: 1 mL/min; and injection volume: 10 *μ*L. The structural characterization of PSG was analyzed by the Fourier transform infrared (FT-IR) spectra (4000–400 cm^−1^).

### 2.3. IBD Mouse Model Establishment and PSG Treatment

**Figure 2:**
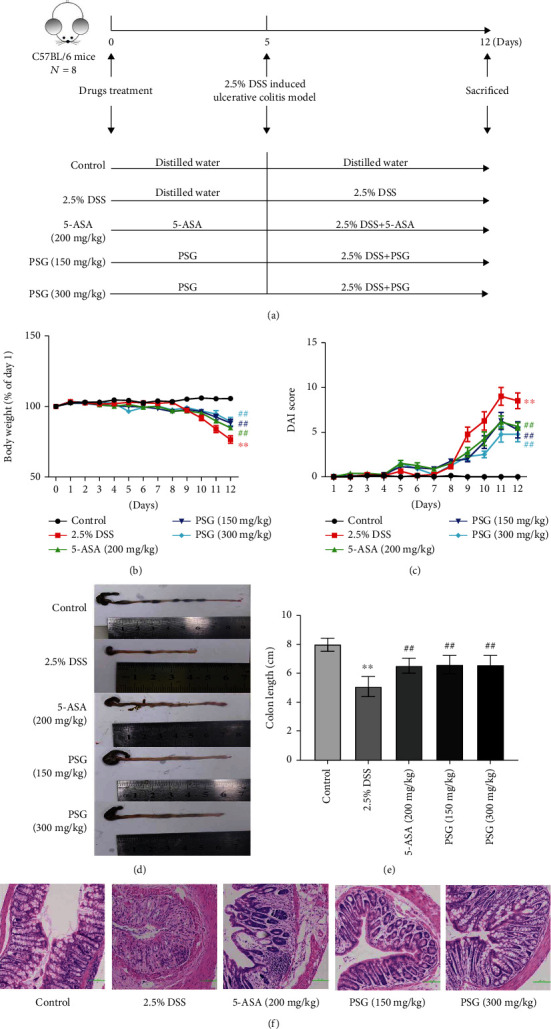
PSG alleviated IBD of mice induced by DSS. (a) Design for PSG in anti-IBD effect on mouse model induced by DSS. (b) Effect of PSG on the bodyweight of mice. (c) Disease activity index of IBD mice. (d) Representative photographs of the colon. (e) Effect of PSG on colon length of IBD mice. (f) Histological analysis for colon tissues by H&E staining. Results were expressed as the mean ± SEM. ^∗^*p* < 0.05 and ^∗∗^*p* < 0.01 vs. the control group; ^#^*p* < 0.05 and ^##^*p* < 0.01 vs. the model group (DSS only).

The experiment was assessed and approved by the Institutional Animal Care and Use Committee of Guangzhou University of TCM, and animal treatments and experimental procedures were carried out strictly following the principle of Laboratory Animal Care and the guidelines of the Guangzhou University of TCM Animal Research Committee (AEWC-2021004). Briefly, C57BL/6J mice (aged 8-10 weeks, ♂) were purchased from Guangdong Medical Laboratory Animal Center (No. 44822700000356). After adaptive feeding (24–25°C, humidity 70–75%, 12 h light/dark) with a standard diet and water, the mice were randomly divided into five groups of 8 animals each, including the control group, 2.5% DSS group, 5-ASA group (200 mg/kg), 2.5% DSS+PSG low-dosage (150 mg/kg) group, and 2.5% DSS+PSG high-dosage (300 mg/kg) group. PSG was given orally for 12 days continuously and 2.5% DSS (*W*/*V*) for 7 days (from day 6 to 12) [23, 24]. The mice were sacrificed (isoflurane inhalation anesthesia) 24 h after PSG treatment was terminated. The whole experimental design is shown in Figure 2(a).

### 2.4. Disease Activity Index (DAI) Evaluation

Disease activity index score was determined according to the body weight loss (no loss recording 0 points, loss 1%~5% recording 1 point, loss 5%~10% recording 2 points, loss 10%~5% recording 3 points, and a loss greater than 15% recording 4 points), fecal viscosity (normal stool recording 0 points, soft stool recording 1 point, loose-wet stool recording 2 points, diarrhea-dry stool recording 3 points, and diarrhea-wet stool recording 4 points), and fecal bleeding (no blood recording 0 points, slight blood recording 1 point, occult blood recording 2 points, bleeding recording 3 points less, and gross bleeding recording 4 points) of IBD mice [25].

### 2.5. Sample Collection

After treatment, the mice were anesthetized to collect the serum and then sacrificed for dissection. Blood was collected from the retroorbital sinus of mice and incubated at room temperature for 1 h. The serum was collected via centrifugation at 3,500 rpm for 10 min. The colon and liver were removed for length measurement and weighed. Parts of colon tissues and liver tissues were fixed in 4% paraformaldehyde (PFA) for hematoxylin and eosin (H&E) staining, and the rest tissues were stored at -80°C.

### 2.6. Biochemical Analysis

Biochemical parameters including ALT and AST in the serum of mice were detected according to the manufacturer's introduction.

### 2.7. ELISA Assay

ELISA assay kits were obtained commercially, and biochemical analysis was performed according to the manufacturer's introduction. Briefly, colon and liver tissues were washed in cold phosphate-buffered saline (PBS, 0.01 M, pH 7.4) and homogenized with 10 times PBS (*V*/*W*). The samples were centrifuged at 5,000 *g* for 10 min at 4°C, and the supernatants were collected for further biochemical analysis.

### 2.8. Western Blot

Colon and liver tissues were lysed with RIPA lysis buffer supplemented with a protease inhibitor and phosphatase inhibitor (Beyotime, China) for 30 min on ice [23]. The samples were collected after centrifugation at 12,000 rpm at 4°C for 10 min, and the protein concentrations were measured by a bicinchoninic acid (BCA) protein assay kit (09282021010, Beyotime, Shanghai, China). Equal amounts of protein (40 *μ*g) were loaded and separated on 8%~15% sodium dodecyl sulfate-polyacrylamide gels, and then transferred onto polyvinylidene difluoride (PVDF) membranes (Merck Millipore Ltd., IPVH00010, Darmstadt, Germany). The membranes were blocked with QuickBlock™ solution (Beyotime, P0252, Shanghai, China) at room temperature for 15 min, washed in PBST (0.1% Tween-20 in PBS), and incubated with primary antibodies of ZO-1, occludin, iNOS, COX2, HO1, NQO1, Keap-1, Nrf2, gasdermin D, NLRP3, caspase 1, ASC, TLR4, MyD88, phospho-NF-*κ*B, NF-*κ*B, phospho-H2AX, SOD2, GPX4, and *β*-actin at 4°C overnight. After being washed with PBST 3 times, the membranes were incubated with secondary antibodies conjugated with horseradish peroxidase (HRP) (1 : 10000) at room temperature for 1.5 h. The membrane blots were detected by an enhanced chemiluminescence (ECL) kit. All gray analyses for protein blots were performed with ImageJ software.

### 2.9. Hematoxylin-Eosin (H&E) Staining

Tissue fixation, dehydration, embedding, and staining were performed according to Reference [26]. The morphological changes in tissues were observed under an optical microscope (Nikon Corporation, 108-6290, Tokyo, Japan), and photos were taken (magnifications, 200x for colon tissues and 100x for liver tissues).

### 2.10. Immunohistochemistry (IHC) Examination

IHC was used to examine the protein expressions of Nrf2 and NLRP3 in the liver tissues and mucin-like glycoprotein 2 (MUC2) in the colon tissues of IBD mice. The procedure was carried out using a formalin-fixed paraffin-embedded tissue according to a reference published previously [27]. The expression changes of MUC2, Nrf2, and NLRP3 were evaluated under an optical microscope (Nikon Corporation, 108-6290, Tokyo, Japan), and photos were taken (magnifications, 400x for colon tissues and 200x for liver tissues).

### 2.11. Statistical Analysis

All data were expressed as the mean ± standard error (SEM). The data were analyzed by *one-way ANOVA*, followed by the post hoc Tukey test. Differences at *p* < 0.05 were considered statistically significant.

## 3. Results

### 3.1. PSG Alleviated IBD of Mice

In our study, the IBD mouse model was established by taking 2.5% DSS (*W*/*V*) orally. As shown in Figures 2(b) and 2(c), the bodyweight loss, diarrhea, and hematochezia of the model group were sharply enhanced after DSS induction, and the DAI scores were significantly higher than those of the control group, indicating the IBD model was established successfully. However, after PSG (150, 300 mg/kg) treatment, diarrhea and rectal bleeding and body weight loss of IBD mice were remarkably alleviated, and DAI scores were significantly reduced. Furthermore, the colon length of the IBD mouse model was significantly reduced compared to the control group, while after PSG treatment for 12 days, the colon length shortening was significantly reversed (Figures 2(d) and 2(e)), indicating the good alleviation of PSG against IBD. Additionally, we performed a histopathological examination of the colon tissues. As a result (Figure 2(f)), compared with the control group, the histopathology of the colon in the DSS induction group was typical, including the colonic mucosa structure remarkably damaged, glands disappeared, and numerous neutrophils and lymphocytes infiltrated in lamina propria and submucosa, indicating that the intestinal barrier was broken and the intestinal permeability was significantly increased. However, after PSG treatment, the colonic mucosa structure was improved, the gland damage and immune cell infiltration were relieved, and goblet cells were visible, which demonstrated the good therapeutic efficacy of PSG in IBD.

### 3.2. PSG Alleviated the Secondary Liver Injury in DSS-Induced IBD of Mice

**Figure 3:**
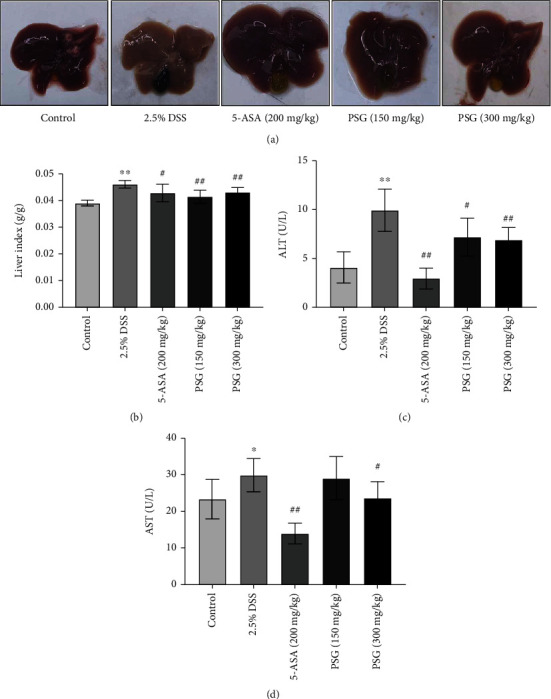
PSG alleviated secondary liver injury (SLI) in DSS-induced IBD of mice. (a) Representative photographs of liver tissues after PSG treatment. (b) Effect of PSG on liver organ index. Effects of PSG on ALT (c) and AST (d) levels in the serum of IBD mice. Results were expressed as the mean ± SEM. ^∗^*p* < 0.05 and ^∗∗^*p* < 0.01 vs. the control group; ^#^*p* < 0.05 and ^##^*p* < 0.01 vs. the model group (DSS only).

Liver dysfunction, including liver steatosis and cholestasis, is a common complication in the occurrence of IBD, during which a series of intestinal toxins would go into the liver through the hepatoenteric circulation and activate the proinflammatory signaling pathways, such as the LPS/TLR4/NF-*κ*B and JAK2/STAT3 pathways, thereby inducing the liver damaged [28]. As shown in our study, the global liver morphology of IBD mice changed to be gray and bloodless, compared with that of the control mice, while the liver morphology of PSG-treated mice recovered to be normal color. The liver index of the IBD mouse model was much higher than that of the control group mice, while the liver indexes of the PSG-treated groups were significantly reduced, indicating the good alleviation of PSG on SLI accompanied with IBD (Figures 3(a) and 3(b)). Furthermore, the levels of ALT and AST, two sensitive markers of acute hepatocyte damage, were also increased in DSS-induced IBD mice, while significantly reversed after PSG treatment (Figures 3(c) and 3(d)). Altogether, our study indicated that PSG could strongly alleviate secondary liver injury in DSS-induced IBD of mice.

### 3.3. PSG Improved the Intestinal Barrier of IBD Mice

**Figure 4:**
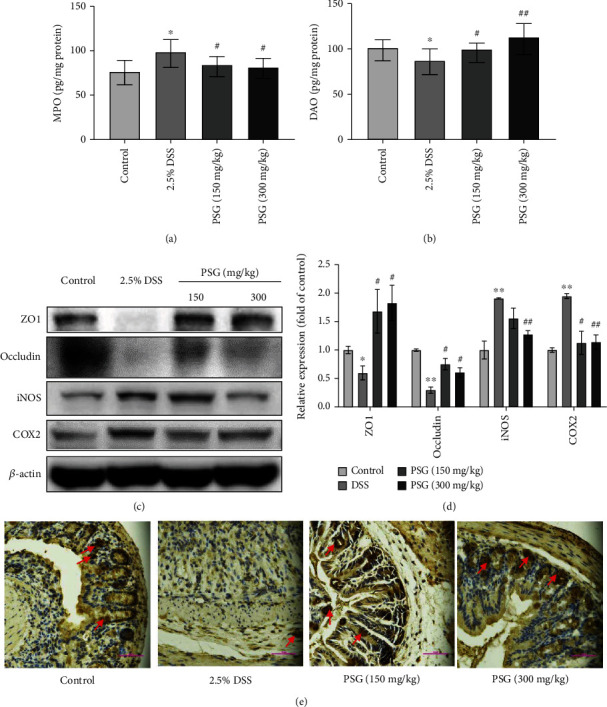
PSG improved the intestinal barrier function of IBD mice. (a) Effect of PSG on MPO level in the colon of IBD mice. (b) Effect of PSG on DAO level in the colon of IBD mice. The expressions of tight junction protein ZO1 and occludin in the colon tissues of IBD mice were evaluated by Western blot assay (c) and quantitative analysis for Western blot results (*n* = 3) (d). (e) The expression of MUC2 in the colon tissues of IBD mice was detected by IHC. Results were expressed as the mean ± SEM. ^∗^*p* < 0.05 and ^∗∗^*p* < 0.01 vs. the control group; ^#^*p* < 0.05 and ^##^*p* < 0.01 vs. the model group (DSS only).

The intestinal barrier is an important function of the intestinal tract, by which the body can effectively prevent the invasion of pathogenic antigens. Hence, protecting or strengthening the intestinal barrier integrity can be recognized as the most important approach for IBD therapy [29]. In this study, we evaluated the changes of myeloperoxidase (MPO), an important marker for intestinal inflammation, and diamine oxidase (DAO), a critical parameter reflecting the integrity and damage degree of the intestinal barrier [30], after PSG treatment. As a result (Figures 4(a) and 4(b)), the levels of MPO increased and DAO decreased after DSS induction, while PSG significantly reversed the changes in MPO and DAO. Furthermore, the expressions of ZO1 and occludin, two important tight junction proteins, remarkably decreased after DSS induction, suggesting the intestinal barrier was damaged seriously. Meanwhile, the levels of inflammatory factors iNOS and COX2 were significantly increased, indicating the occurrence of intestinal inflammation. However, after PSG treatment, the expressions of ZO1 and occludin were significantly increased, while those of iNOS and COX2 were decreased (Figures 4(c) and 4(d)), showing the good efficacy of PSG in protecting the intestinal barrier from the damage of IBD mice. Additionally, we examined the change of MUC2, an important protein that is secreted by the goblet cells in the intestinal tract and reflects the occurrence and healing of IBD, via IHC assay. The results showed that PSG reversed the reduction of MUC2 in DSS-induced IBD mice (Figure 4(e)). These data indicate the strong prevention of PSG against the permeability of the intestinal barrier in IBD mice.

### 3.4. PSG Protected from SLI via Suppressing the LPS/TLR4/MyD88/NF-*κ*B Pathway in DSS-Induced IBD Mice

**Figure 5:**
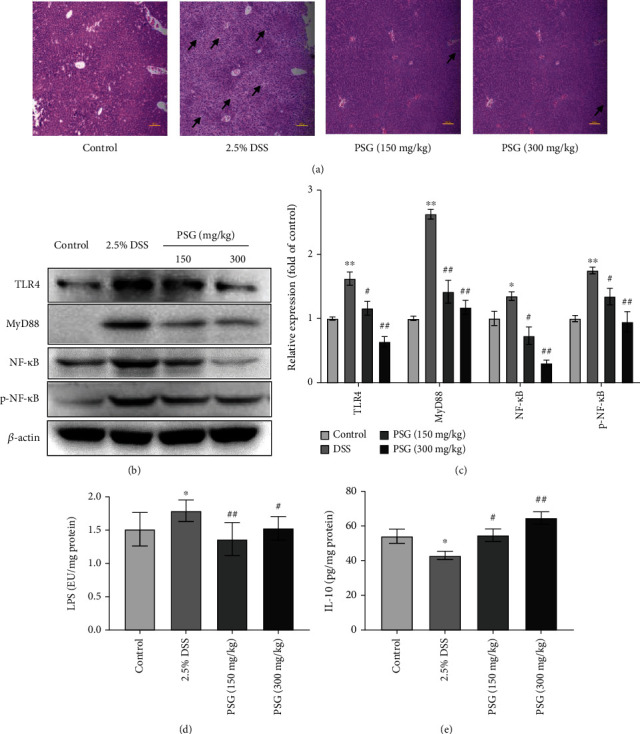
PSG suppressed SLI via inhibiting the LPS/TLR4/MyD88/NF-*κ*B pathway in DSS-induced IBD mice. (a) Histological analysis for liver tissues by H&E staining. Protein expressions of the TLR4/MyD88/NF-*κ*B pathway in the liver tissues were determined by Western blot assay (b) and quantitative analysis for Western blot results (*n* = 3) (c). Effects of PSG on LPS (d) and IL-10 (e) in the liver tissues of IBD mice. Results were expressed as mean ± SEM. ^∗^*p* < 0.05 and ^∗∗^*p* < 0.01 vs. the control group; ^#^*p* < 0.05 and ^##^*p* < 0.01 vs. the model group (DSS only).

In the IBD model, the leakage of the intestinal tract may cause excessive lipopolysaccharide (LPS) shifting into the liver through the portal vein circulation and promoting a series of proinflammatory cytokines to release, thereby inducing chronic inflammatory response via activating the proinflammatory signaling pathway such as the TLR4/MyD88/NF-*κ*B pathway, and a long-term continuous chronic inflammation can trigger secondary liver injury and hepatitis [31]. Interleukin-10 (IL-10), an important regulatory cytokine inhibiting the antigen presentation and inflammatory cytokines release, is considered to be a good candidate for chronic IBD therapy [32]. According to pathological analysis results, it was obvious that the liver cells of the DSS group were disorganized, with blurred boundaries, severe steatosis, and inflammatory infiltration. After PSG treatment, the pathological changes in liver tissues were significantly improved (Figure 5(a)). Then, we evaluated the effect of PSG on the TLR4/MyD88/NF-*κ*B pathway of liver tissues via Western blot assay. The results showed that the levels of TLR4, MyD88, phospho-NF-*κ*B, and NF-*κ*B in the livers were remarkably enhanced in the DSS-induced IBD model group, while those in the PSG-treated groups were significantly reversed (Figures 5(b) and 5(c)). Additionally, we evaluated the levels of LPS and IL-10 in liver tissues of IBD mice via the ELISA assay. As a result (Figures 5(d) and 5(e)), DSS significantly increased the level of LPS and reduced the level of IL-10 in the liver tissues of mice. However, after PSG treatment, the levels of LPS and IL-10 were significantly reversed, indicating the good suppression of PSG in the secondary liver injury of IBD mice. Altogether, these data suggested that PSG prevented the intestinal tract from leakage of IBD mice induced by DSS, and suppressed the IBD-associated secondary liver injury via inhibiting the LPS/TLR4/MyD88/NF-*κ*B signaling pathway. However, the detailed pharmacological mechanism is still elusive.

### 3.5. PSG Alleviated SLI of IBD Mice via Suppressing Pyroptosis and Oxidative Damage

**Figure 6:**
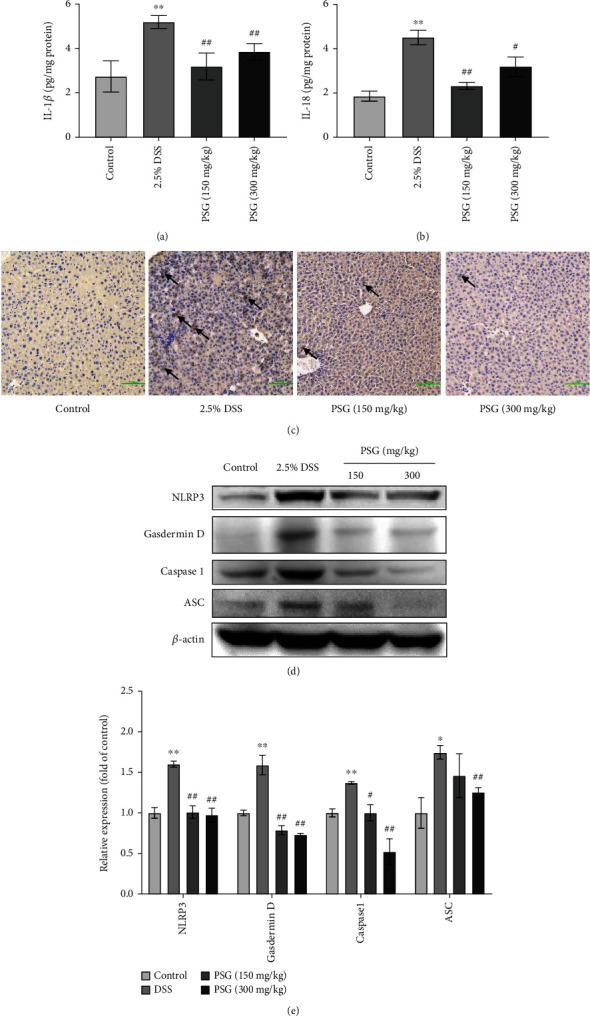
PSG suppressed pyroptosis of the liver tissues in IBD mice. Effect of PSG on the levels of IL-1*β* (a) and IL-18 (b) in the liver tissues of IBD mice. (c) Protein expression of NLRP3 in the liver tissues was detected by IHC. Protein expressions of NLRP3, gasdermin D, caspase 1, and ASC in the liver tissues were determined by Western blot assay (d) and quantitative analysis for Western blot results (*n* = 3) (e). Results were expressed as the mean ± SEM. ^∗^*p* < 0.05^∗∗^*p* < 0.01 vs. the control group; ^#^*p* < 0.05 and ^##^*p* < 0.01 vs. the model group (DSS only).

In our study, the effects of PSG on pyroptosis and inflammation of IBD-associated SLI mice were evaluated via Western blot, ELISA, and IHC assays. The results of the ELISA assay showed that PSG significantly reduced the high levels of IL-1*β* and IL-18 in the livers of DSS-induced IBD mice (Figures 6(a) and 6(b)), and IHC staining indicated that DSS increased the expression of NLRP3 while PSG reversed it (Figure 6(c)). Furthermore, the Western blot assay also showed that the levels of NLRP3, gasdermin D, caspase 1, and ASC in the livers were increased after DSS induction, while those of the PSG-treated groups were significantly reduced (Figures 6(d) and 6(e)), suggesting that PSG could significantly inhibit pyroptosis and inflammation of liver tissues induced by DSS.

**Figure 7:**
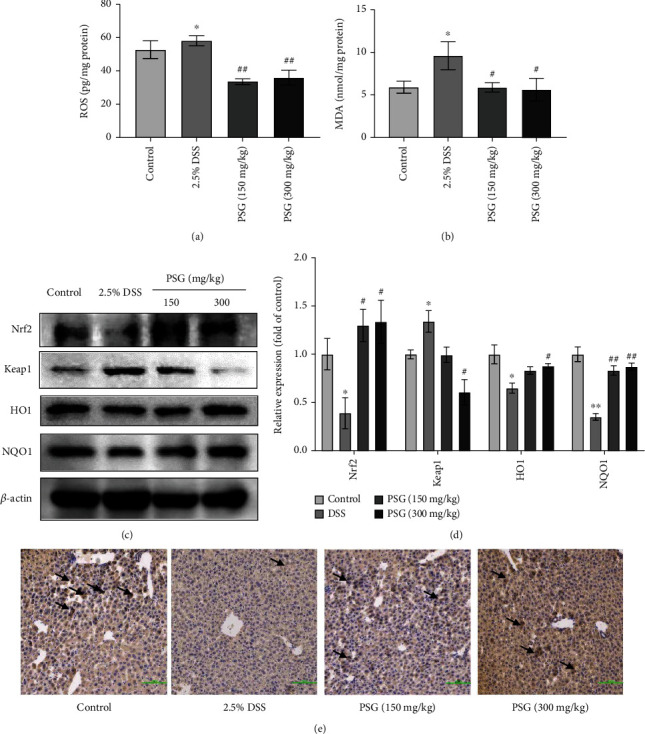
PSG inhibited oxidative stress of the liver tissues in IBD mice. Effect of PSG on the levels of ROS (a) and MDA (b) in the liver tissues of IBD mice. The protein expressions of the Nrf2/Keap1/HO1/NQO1 pathway in the liver tissues were determined by Western blot assay (c) and quantitative analysis for Western blot results (*n* = 3) (d). (e) The protein expression of Nrf2 in the liver tissues was detected by IHC. Results were expressed as the mean ± SEM. ^∗^*p* < 0.05 and ^∗∗^*p* < 0.01 vs. the control group; ^#^*p* < 0.05 and ^##^*p* < 0.01 vs. the model group (DSS only).

We also evaluated the effect of PSG on the oxidative stress of SLI mice. As a result, PSG significantly reduced the high levels of ROS and MDA in the liver tissues of DSS-induced IBD mice (Figures 7(a) and 7(b)). Western blot assay showed that DSS increased the level of Keap1 and decreased the levels of Nrf2, HO1, and NQO1, while PSG significantly reversed the effects (Figures 7(c) and 7(d)), suggesting that PSG alleviated the oxidative stress of liver tissues in IBD mice by regulating the Keap1/Nrf2/ARE/HO-1 signaling pathway. Furthermore, an IHC assay was used to detect the expression of Nrf2, the critical factor of the Keap1/Nrf2/ARE/HO-1 pathway, in the liver tissues of IBD mice, and the results showed that PSG enhanced the expression of Nrf2 in DSS-induced IBD mice (Figure 7(e)).

**Figure 8:**
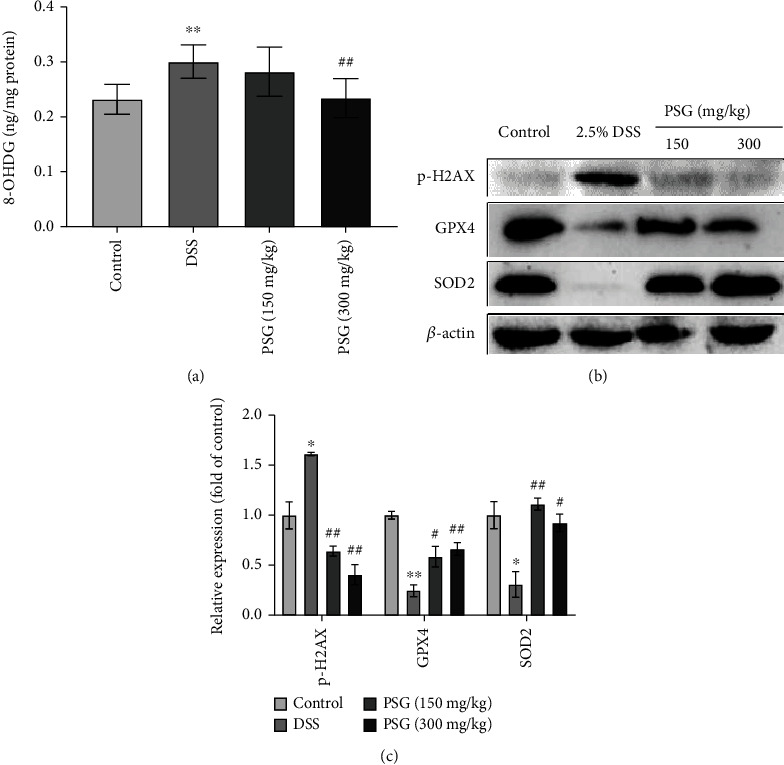
PSG alleviated oxidative damage of the liver tissues in IBD mice. (a) Effect of PSG on the level of 8-OHDG in the liver tissues of IBD mice. The protein expressions of phospho-H2AX, GPX4, and SOD2 in the liver tissues were determined by Western blot assay (b) and quantitative analysis for Western blot results (*n* = 3) (c). Results were expressed as the mean ± SEM. ^∗^*p* < 0.05 and ^∗∗^*p* < 0.01 vs. the control group; ^#^*p* < 0.05 and ^##^*p* < 0.01 vs. the model group (DSS only).

The long cause of oxidative stress would enhance the level of oxidation products such as 8-OHDG via triggering a series of oxidative pathways, resulting in tissue damage severely [33]. In this study, we further investigated the influence of PSG on oxidative damage of liver tissues in SLI mice. The results of the ELISA assay showed that PSG significantly decreased the level of 8-OHDG (Figure 8(a)) of SLI mice, and the Western blot assay indicated that DSS increased the level of PSG phospho-H2AX and decreased the levels of GPX4 and SOD2 in the liver tissues of SLI mice, while PSG dramatically reversed the effects (Figures 8(a)–8(c)), suggesting the good suppressed efficacy of PSG on oxidative damage of SLI mice.

In all, our findings suggested that PSG alleviated IBD and IBD-associated secondary liver injury via suppressing pyroptosis and oxidative damage in mice.

### 3.6. Characterization of PSG

**Figure 9:**
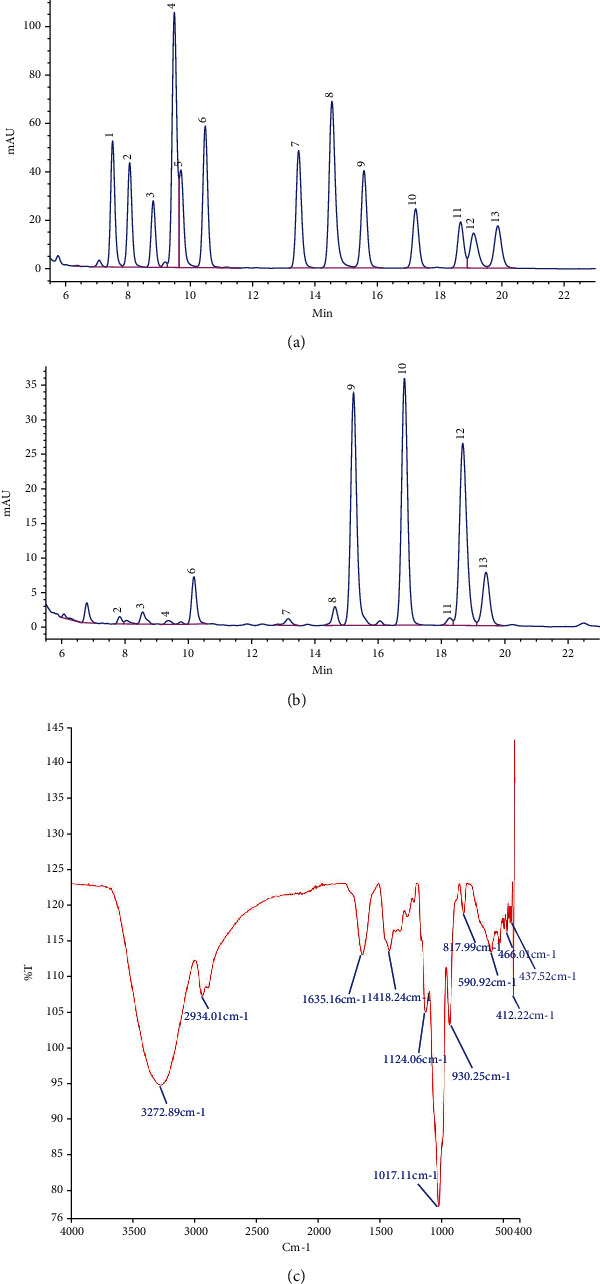
Characterization of the polysaccharides from garlic (PSG). (a) A standard mixture of monosaccharides (1: guluronic acid, 2: mannuronic acid, 3: mannose, 4: glucosamine, 5: ribose, 6: rhamnose, 7: glucuronic acid, 8: galactosamine, 9: galacturonic acid, 10: glucose, 11: xylose, 12: galactose, 13: arabinose, and 14: L-fucose). (b) Analysis of monosaccharide composition of PSG. (c) FT-IR spectra of PSG.

PSG was determined to consist of 80.64% total polysaccharide content (*W*/*W*) detected by the phenol-sulfuric acid method and 10.14% protein content (*W*/*W*) detected by a protein assay kit. The average molecular weight of PSG was calculated to be 8242 Da based on the GPC analysis. The compositions of monosaccharides and uronic acid in PSG are shown in Figures 9(a) and 9(b), including mannuronic acid (0.092%), mannose (0.323%), glucosamine (0.026%), rhamnose (0.474%), glucuronic acid (0.107%), galactosamine (0.137%), galacturonic acid (3.582%), glucose (5.913%), xylose (0.209%), galactose (6.738%), and arabinose (1.755%). The FT-IR spectrum of PSG is shown in Figure 9(c), including the absorption at 3272.89 cm^−1^ standing for the O-H stretching vibration, the absorption peak at 2934.01 cm^−1^ being the C-H stretching vibration which is the characteristic absorption peak of polysaccharides, the absorption at 1635.16 cm^−1^ representing the stretching vibration of C=O, the absorption peak at 1124.06 cm^−1^ showing the stretching vibration of C-O-C, indicating that PSG may have a pyran ring, and the absorption peak at 930.25 cm^−1^ indicating that the glycosidic bond would be in the *β* configuration.

## 4. Discussion

IBD, a group of chronic intestinal nonspecific diseases with unknown etiology and pathogenesis [34], mainly consists of ulcerative colitis (UC) and Crohn's disease (CD) [35]. It is a multisystem disease with extraintestinal symptoms that may involve the skin, bone, kidney, liver, and so on [10, 12]. The prolonged course of IBD makes it difficult to cure, which would lead to a decline in patients' quality of life and working efficiency [36]. It is reported that IBD acts as a high-risk factor for colorectal cancer development [37]. Various hepatobiliary diseases are also closely related to IBD, among which hepatitis is the most common complication, with incidence rates of 1.5~39.5% in CD and 1.5~55% in UC [38]. In the pathological status of IBD, with the intestinal mucosal barrier function impaired, the intestinal permeability increased, and the overgrown bacteria together with their products, such as the endotoxin and proinflammatory factors, would enter the liver through the portal vein, thereby activating the nonspecific immune system of the liver and induce a large number of inflammatory cytokines and chemokines to secrete, causing or aggravating the inflammatory response of the liver, which would lead to the occurrence of hepatitis, liver cirrhosis, or even liver cancer [39, 40].

Treatments used in the clinic mainly include aminosalicylic acid, hormone, azathioprine, cyclosporine, thalidomide, infliximab, adalimumab, vedolizumab, and selective leukocyte adsorption therapy [41–43]. However, the efficacies of these treatments are not satisfactory as some of them may induce significant side effects for a long-time use and some of them only target one kind of cytokines. More importantly, most of these drugs cannot alleviate SLI sufficiently when treating IBD. Hence, discovering new alternative agents with high efficacy, low toxicity, multitargets, and benefit in alleviating complications of IBD is largely demanded [44, 45]. It is reported that the polysaccharides from wild jujube sarcocarp suppressed TNBS-induced IBD in rats by reducing the levels of TNF-*α*, IL-1*β*, IL-6, and MPO mediated by activating the AMPK pathway [46]. In this study, we found that PSG could significantly alleviate DSS-induced IBD by ameliorating the colon length loss, bodyweight loss, diarrhea, and bleeding feces and inhibiting the hepatic steatosis and cholestasis, reducing the high levels of ALT and AST in the serum of UC mice, which suggested that PSG can be regarded as a therapeutic candidate for IBD and IBD accompanied with SLI.

The intestinal barrier is the most important line of defense in the body, which can effectively prevent harmful substances such as bacteria and endotoxin from entering the blood and organs, and the hepatoenteric circulation acts a critical role in the crosstalk between the intestinal tract and liver. LPS, a classic endotoxin entering the liver through the gut-liver axis, would activate the Toll-like receptor 4- (TLR4-) dependent proinflammatory pathway and then trigger the inflammatory response or fibrotic hyperplasia of the liver [28]. Therefore, drugs that can protect the intestinal barrier from damage and modulate the hepatoenteric circulation are regarded as prospected candidates for treating IBD and SLI. Chen et al. reported that the natural compound monotropein could protect against secondary liver injury caused by chronic colitis [47]. In our study, PSG was found to protect the intestinal barrier by increasing the expressions of tight junction protein ZO1 and occludin of IBD mice, reduce the level of LPS in liver tissues, and suppress the LPS/TLR4/MyD88/NF-*κ*B signaling pathway, implying that PSG could protect the colon from damage, improve the intestinal permeability, and also found to prevent IBD-associated liver damage through regulating the gut-liver axis.

It is reported that genetic background change, environmental factor influence, and microbiota disorder are usually implicated during IBD evolution [48]. Inflammation is a typical characteristic of IBD, and a series of proinflammatory cytokines, such as TNF-*α*, IL-1*β*, and IL-6, are overproduced. Pyroptosis, known as inflammatory necrosis, is a type of programmed cell death, in which cells swell until their membranes rupture, leading to the release of cell contents and activation of an intense inflammatory response [49]. It is reported that pyroptosis is widely involved in the occurrence, development, and prognosis of various digestive diseases, such as hepatitis, cirrhosis, IBD, and gastrointestinal tumors [50, 51]. During pyroptosis, NLRP3 inflammasome is considered to take charge of the processing and secreting of mature IL-1*β*, activating of caspase 1 mediated by interaction with apoptosis-associated speck-like protein (ASC) [52], and the pattern recognition receptors (PRR) would bind to the precursor of caspase 1 through ASC and NLRP3 to form a complex, which would activate gasdermin D and then promote the release of inflammatory factors, resulting in pyroptosis [53]. Li et al. reported that blocking gasdermin D would protect hepatic ischemia-reperfusion from injury [54] and the natural product irisin could suppress liver injury by inhibiting pyroptosis [55]. In our study, we evaluated the effect of PSG on inflammation and pyroptosis of liver tissues in IBD mice. As a result, the levels of IL-18 and IL-1*β* were significantly reduced, and the protein expressions of NLRP3, gasdermin D, caspase 1, and ASC were also significantly decreased after PSG treatment, suggesting the good suppression of PSG on pyroptosis in treating SLI of the IBD mouse model.

Oxidative stress, a state of imbalance between oxidation and antioxidant, would promote a large number of oxidative intermediates to produce, which would lead to the secretion of protease and inflammatory infiltration of neutrophils, and the large quantities of oxidative-active molecules accumulating in the body would cause cells and tissues damaged seriously [22, 56]. It was also reported that oxidative stress could promote the activation of pyroptosis in liver injury [57]. Nrf2, a critical gene in regulating the expressions of antioxidant genes, is very important for the body's response to oxidative stress [58]. It is located in the cytoplasm and binds with Keap1 in an inactive state and can be continuously degraded by the ubiquitin-proteasome [59]. When stimulated by the oxidative stress signal, Nrf2 immediately uncouples with the allosteric Keap1, translocated into the nucleus to form a heterodimer, and then binds to the corresponding sites of antioxidant response element (ARE) to initiate the transcription and translation of phase II detoxification enzyme and antioxidant protein [60–62]. Yu et al. reported that oxidative stress was one of the major pathologies of liver injury, and baicalein, a natural compound isolated from *Scutellaria baicalensis* G., could protect the liver from injury via suppressing oxidative stress [63]. In the present study, PSG was found to significantly reduce the levels of ROS and MDA, increase the expressions of Nrf2, HO1, and NQO1, and decrease the expression of Keap1 in liver tissues, indicating the good efficacy of PSG against SLI via suppressing oxidative stress. Overaccumulation of ROS will cause 8-OHDG increased and membrane lipid peroxidation, resulting in DNA or tissue damage, and even carcinogenic genes activated [64]. GSH deficiency can lead to the inactivation of GPX4, the important protein for reducing the toxicity of lipid peroxides and maintaining the homeostasis of membrane structures [65]. In our study, DSS induced high levels of 8-OHDG and phospho-H2AX, and a low level of SOD2, suggesting the presence of oxidative damage in SLI mice. PSG significantly reversed the liver injury, decreased the levels of 8-OHDG and phospho-H2AX, and increased the expression of SOD2, which suggested that PSG could alleviate SLI in IBD mice via suppressing oxidative damage to liver tissues.

**Figure 10:**
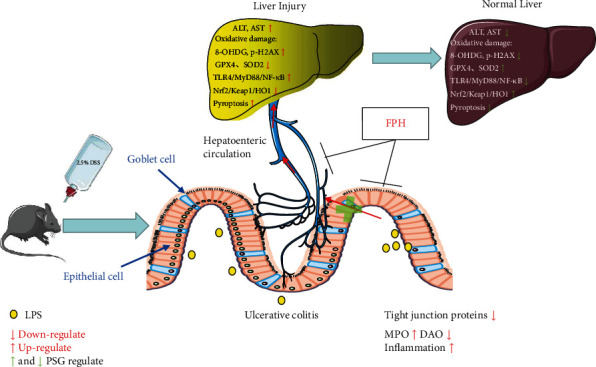
Proposed mechanism of PSG in treating DSS-induced IBD and SLI of mice. Polysaccharides of garlic (PSG); interleukin-1*β* (IL-1*β*); interleukin 18 (IL-18); reactive oxygen species (ROS), malondialdehyde (MDA), alanine aminotransferase (ALT), aspartate aminotransferase (AST), Toll-like receptor 4 (TLR4), myeloid differentiation factor 88 (MyD88), nuclear factor-k-gene binding (NF-*κ*B), nuclear factor E2-related factor 2 (Nrf2), Kelch-like ECH associated protein 1 (Keap1), heme oxygenase 1 (HO1), myeloperoxidase (MPO), diamine oxidase (DAO), lipopolysaccharide (LPS), upregulation (↑), and downregulation (↓).

Altogether, this study demonstrated that PSG could not only inhibit IBD via inhibiting the LPS/TLR4/MyD88/NF-*κ*B pathway but also protect secondary liver injury via suppressing pyroptosis and oxidative injury (Figure 10).

## 5. Conclusions

These data indicated that PSG exerted an excellent inhibition on DSS-induced IBD by strengthening the intestinal barrier of mice and strongly alleviated SLI via inhibiting pyroptosis and oxidative injury. Our study for the first time reveals the alleviation of PSG in treating inflammatory bowel disease accompanied by secondary liver injury in mice, as well as uncovers its underlying mechanism. The data would provide a better understanding of PSG in treating IBD and secondary liver injury, suggesting that the garlic polysaccharides can be enlarged to treat IBD and secondary liver injury in humans.

## Data Availability

Data in this study are available from the corresponding authors upon request.
